# Thermal deprotection: a sustainable and efficient strategy for synthesising α-polylysine adsorbents†

**DOI:** 10.1039/d5ra00641d

**Published:** 2025-05-23

**Authors:** Xuchen Jin, Paul D. Thornton

**Affiliations:** a School of Chemistry, University of Leeds Leeds LS2 9JT UK; b School of Design, University of Leeds Leeds LS2 9JT UK p.d.thornton@leeds.ac.uk; c Leeds Institute of Textiles and Colour (LITAC), University of Leeds Leeds LS2 9JT UK

## Abstract

α-Polylysine (PLys) is a versatile, renewable, and biodegradable polymer with extensive amine functionality and water-solubility, making it an ideal candidate for critical applications such as heavy metal adsorption and beyond. However, conventional synthesis of linear PLys relies on toxic reagents for side-chain deprotection, raising environmental and safety concerns that hinder its commercial scalability and sustainability. In this work, we introduce a groundbreaking, environmentally friendly method for PLys production using thermal deprotection of fluorenylmethyloxycarbonyl (Fmoc)-protected PLys. This innovative approach eliminates the need for hazardous deprotection agents, offering a greener and more cost-effective alternative. Beyond the synthesis of homopolymeric PLys, we extend this method to create α-polylysine-*b*-poly(ethylene glycol) (PEG-*b*-PLys) block copolymers using thermal deprotection, showcasing their superior performance in removing Pb^2+^ ions from aqueous solutions. Our results not only advance sustainable polymer synthesis but also demonstrate the potential of thermally driven deprotection to revolutionise wastewater treatment technologies and expand the scope of PLys applications in environmental remediation and other critical industries.

## Abstract 2

Polylysine creation by thermal deprotection offers a novel, green, and straightforward route to effective lead adsorption.

## Graphical abstract

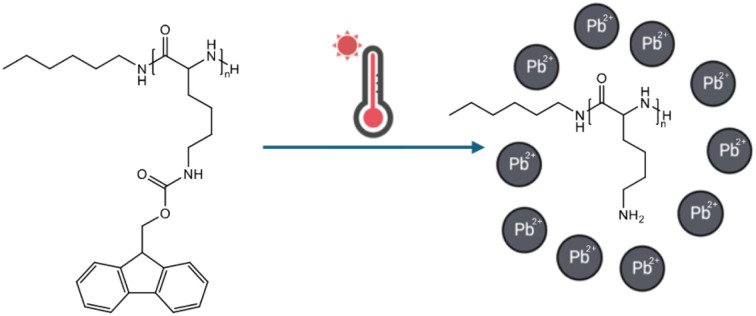

## 1. Introduction

Poly(amino acids) are a class of bio-renewable and biodegradable polymers that derive their versatile functionalities from amino acid repeat units.^1–3^ With 20 proteogenic amino acids available, these polymers can host diverse functional groups, such as amino, thiol, and carboxyl.^4^ This inherent chemical diversity enables applications in drug delivery,^5–7^ tissue engineering,^8–10^ and biosensing.^11–13^ Additionally, poly(amino acids) that possess alkene and alkyne functionalities have been produced and allow straightforward and effective polymer modification *via* click chemistry, further broadening their potential uses.^14^ These properties, combined with their environmental compatibility, position poly(amino acids) as promising alternatives to non-biodegradable, petroleum-derived polymers, provided that sustainable methods of polymer synthesis are employed.

Among poly(amino acids), α-polylysine (PLys) has emerged as a particularly promising candidate due to its unique combination of water solubility, biodegradability, and rich primary amine functionality.^15^ These attributes make PLys highly versatile, with applications ranging from antimicrobial coatings and protein release systems to heavy metal adsorption.^16,17^ PLys has also shown great promise in advanced biomedical applications. For instance, research by the Heise group demonstrated the creation of high molecular weight (MW = 765 000 Da) PLys star polymers, capable of encapsulating and delivering plasmid DNA for gene therapy.^18^ Additionally, PLys-based materials have been effectively used to adsorb heavy metal ions such as CrO_4_^2−^ and Cu^2+^ from aqueous solutions, a property attributed to the strong chelation interactions between the primary amine groups of PLys and the target metal ions.^19^

Heavy metal contamination, particularly lead (Pb^2+^), represents a pressing environmental challenge. Lead, commonly found in industrial effluents such as those from lead-acid batteries, poses severe risks even at low concentrations.^20,21^ It has been linked to significant ecological damage and human health problems, including cognitive impairment, kidney damage, and increased cancer risks.^22,23^ Various methods for Pb^2+^ removal from wastewater have been developed, including chemical precipitation, ion exchange, flocculation, membrane filtration, electrochemical treatments, and bio-adsorption.^24^ Among these, bio-adsorption stands out for its simplicity, cost-effectiveness, and ability to operate efficiently across a wide pH range.^25,26^ Polymers such as PLys, with abundant pendant primary amine groups, are particularly well-suited for this application due to their strong metal-binding capabilities.

Despite its potential for widespread application, the commercial viability of PLys is hindered by challenges associated with its synthesis. The conventional production method for PLys relies on *N*-carboxyanhydride (NCA) ring-opening polymerisation (ROP), which proceeds in three main steps: (1) the synthesis of an NCA monomer from side-chain protected lysine, (2) polymerisation of the NCA monomer to yield side-chain protected PLys, and (3) deprotection of the side-chain groups to liberate the amino functionality.^27^ While this method is more efficient than solid-phase synthesis and more controlled than polycondensation, it requires side-chain protection to prevent premature NCA ring-opening, and all methods require side-chain protection to prevent the formation of branched polymers.

The final PLys deprotection step is particularly problematic as it conventionally involves highly toxic reagents.^28^ For instance, benzyloxycarbonyl(z) protecting groups are commonly used for Lys NCA synthesis, but PLys(z) deprotection requires hazardous reagents such as hydrogen bromide in strongly acidic solutions for cleavage, leading to safety risks.^29^ Alternative protecting groups such as *t*-butyloxycarbonyl (Boc) and fluorenylmethyloxycarbonyl (Fmoc) may also be used, but they still require strong acidic or basic agents for their removal.^29,30^ Even efforts to use less toxic reagents, such as piperazine with 2% 1,8-diazabicyclo[5.4.0]undec-7-ene (DBU) for Fmoc deprotection, face economic and environmental barriers that limit scalability.^31^ The Boc group may also be prematurely cleaved during conventional NCA synthesis by the HCl produced, causing premature polymerisation in the monomer synthesis step.^32^

To address these limitations, we explored the use of thermal deprotection as a sustainable alternative. This approach eliminates the need for hazardous reagents, relying instead on heat to remove Fmoc groups from PLys in dimethyl sulfoxide (DMSO), which can be readily recovered by simple distillation. The method not only simplifies PLys synthesis but also aligns with the principles of green chemistry, reducing the environmental impact and improving the safety and scalability of the process. The use of piperidine is eliminated and DMF and is replaced with DMSO which, whilst not a completely green solvent, has lower relative toxicity in comparison to DMF.^32,33^

We report the synthesis and characterisation of both homopolymeric PLys and α-polylysine-*b*-poly(ethylene glycol) (PEG-*b*-PLys) block copolymers produced using thermal deprotection. The efficacy of these polymers for adsorbing Pb^2+^ ions from aqueous solutions was assessed to determine their potential as efficient and sustainable materials for wastewater treatment. By addressing the dual challenges of hazardous deprotection in PLys synthesis and the need for effective heavy metal remediation, this work underscores the feasibility of thermally driven PLys production as a safer, greener, and more cost-effective pathway for developing high-performance bio-based polymers.

## 2. Results and discussion

The commonly used benzyloxycarbonyl (*z*) protecting group can be used to produce both Lys(z) NCA and PPLy(z) by NCA ROP effectively. However, the z group is thermally stable and thus unsuitable for deprotection in the absence of harsh, acidic, chemicals.^34^ In contrast, the thermal deprotection of Fmoc groups in dimethyl sulfoxide (DMSO) at 120 °C has been reported to achieve over 96% cleavage from FmocProOH and FmocLys (BOC)OH,^35^ prompting the question: can this methodology be extended to macromolecular poly(amino acid) deprotection? If successful, this would eliminate the need for toxic reagents, significantly enhancing the environmental sustainability and cost-effectiveness of PLys production, enabling its enhanced application for instance as a scavenger for metal ions.

**Scheme 1:**
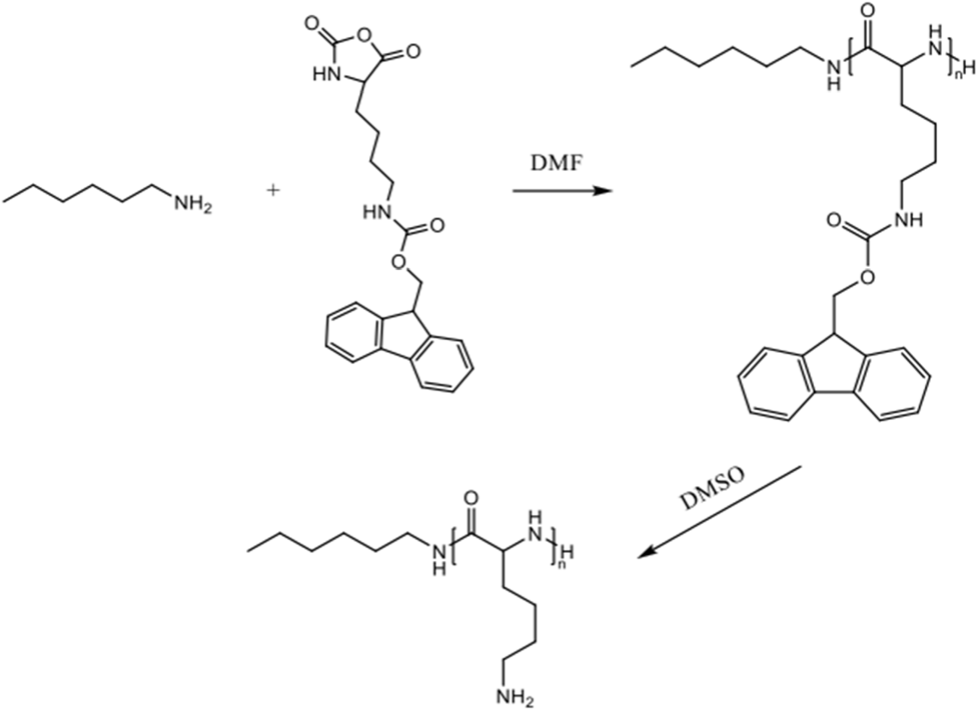
The synthesis of PLys(Fmoc).

The synthetic pathway employed in this study consisted of three steps (Scheme 1): (1) synthesis of Lys(Fmoc)-NCA, (2) polymerisation of Lys(Fmoc)-NCA to yield PLys(Fmoc), and (3) thermal deprotection of PLys(Fmoc) to produce PLys. Additionally, a block copolymer, mPEG-*b*-PLys, was targeted to determine the versatility of thermal deprotection for Fmoc cleavage from larger amphiphilic block copolymers. The inclusion of a PEG block was expected to improve polymer solubility or dispersion in aqueous environments, particularly when high levels of Lys-metal coordination occurred in the target application of Pb^2+^ adsorption.

### 2.1 Synthesis and characterisation

**Fig. 1:**
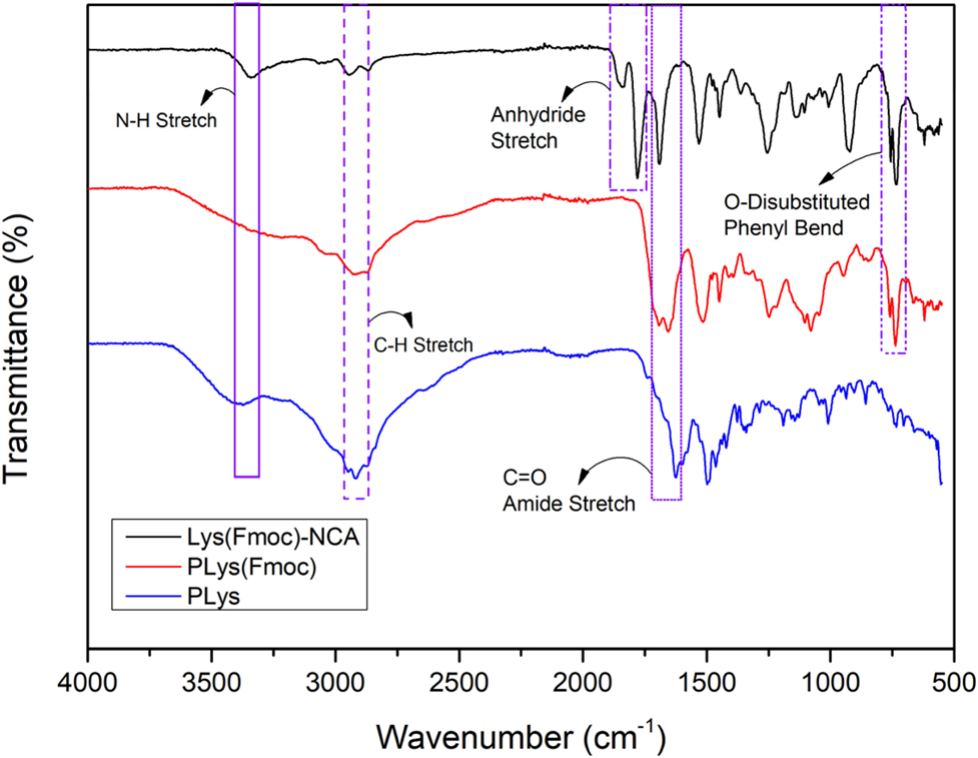
FTIR spectrum corresponding to Lys(Fmoc)-NCA, PLys(Fmoc), and PLys.

Lys(Fmoc)-NCA was produced as an off-white powder.^36^^1^H NMR spectroscopy analysis confirmed successful synthesis;^37^ the peak at 9.10 ppm corresponds to the NH group within the five-membered ring, peaks in the range of 7.94–7.31 ppm are associated with the aromatic protons of the Fmoc protecting groups, the CH_2_ protons in the Fmoc structure are represented by a peak at 4.47–4.39 ppm, and the peak between 1.78–1.10 ppm is attributed to the protons of the CH_2_ groups on the side chain (Fig. S1†). FTIR spectroscopy further confirmed successful NCA synthesis^38^ (Fig. 1), with the spectrum featuring peaks at 1842 cm^−1^ and 1780 cm^−1^ that represent the C=O bonds of the NCA. The peaks at approximately 757 cm^−1^ and 735 cm^−1^ (bend vibrations of O-disubstituted benzyl ring), combined with the peak at 1690 cm^−1^ (C=O from acylamino), corresponded to the Fmoc protecting groups.

Hexylamine was chosen as the initiator for Lys(Fmoc) NCA polymerisation due to its easily identifiable alkyl chain which allows precise quantification of the degree of polymerisation by ^1^H NMR spectroscopy (Scheme 1). The corresponding ^1^H NMR spectrum revealed a peak at 0.87 ppm that represents the terminal methyl group of hexylamine^39^ (Fig. S2†). The peaks between 7.00–8.00 ppm represent the aromatic protons of the Fmoc group. Comparison of the integrals representative of both groups provides a strong indication of the average degree of polymerisation as one group (hexylamine methyl protons) is provided by the initiator whilst the other group is provided by the polymer repeat unit (Fmoc aromatic protons). Complete NCA ring-opening was confirmed by the disappearance of anhydride stretches at 1842 cm^−1^ and 1780 cm^−1^ in the FTIR spectrum (Fig. 1). Advanced Polymer Chromatography (APC) analysis of the PLys(Fmoc) produced indicated a number average molecular weight (*M*_n_) of 16 000 Da and a dispersity of 1.06, corresponding to a monodisperse polymer of 47 repeat units on average. These results confirm that both Lys(Fmoc) NCA and PLys(Fmoc) syntheses are efficient and provide a viable route to PLys.

**Scheme 2:**
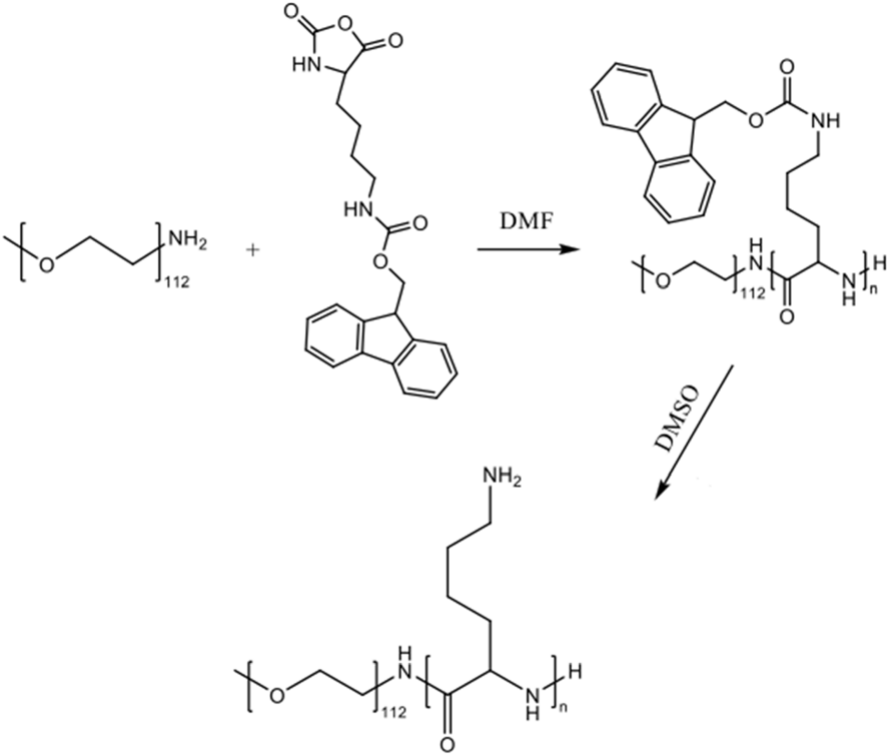
The synthesis of PEG-b-PLys(Fmoc).

PLys is an excellent candidate to be used for heavy metal adsorption. However, metal binding to the primary amine sites of the polymer may reduce polymer solubility in aqueous solutions, potentially leading to aggregation and reduced activity. To ensure that PLys remains soluble, or at least dispersed, in aqueous solution, the diblock copolymer methoxyPEG-*b*-PLys (mPEG-*b*-PLys) was created. The intended role of the PEG block is to ensure polymer solubility even at large levels of Lys-metal coordination as it is not anticipated to participate in metal binding. mPEG-amine has proven to be a very effective initiator for the creation of amphiphilic PEG-*b*-poly(amino acid) block copolymers that are highly promising for controlled release applications, including their potential use as drug delivery vehicles.^40^ Although mPEG-b-PLys(Fmoc) is an amphiphilic block copolymer that may be suited for nanoparticle formation and controlled release applications, the focus of this research is on the creation of polymers for Pb^2+^ adsorption and so the doubly hydrophilic block copolymer mPEG-*b*-PLys was created (Scheme 2).

**Fig. 2:**
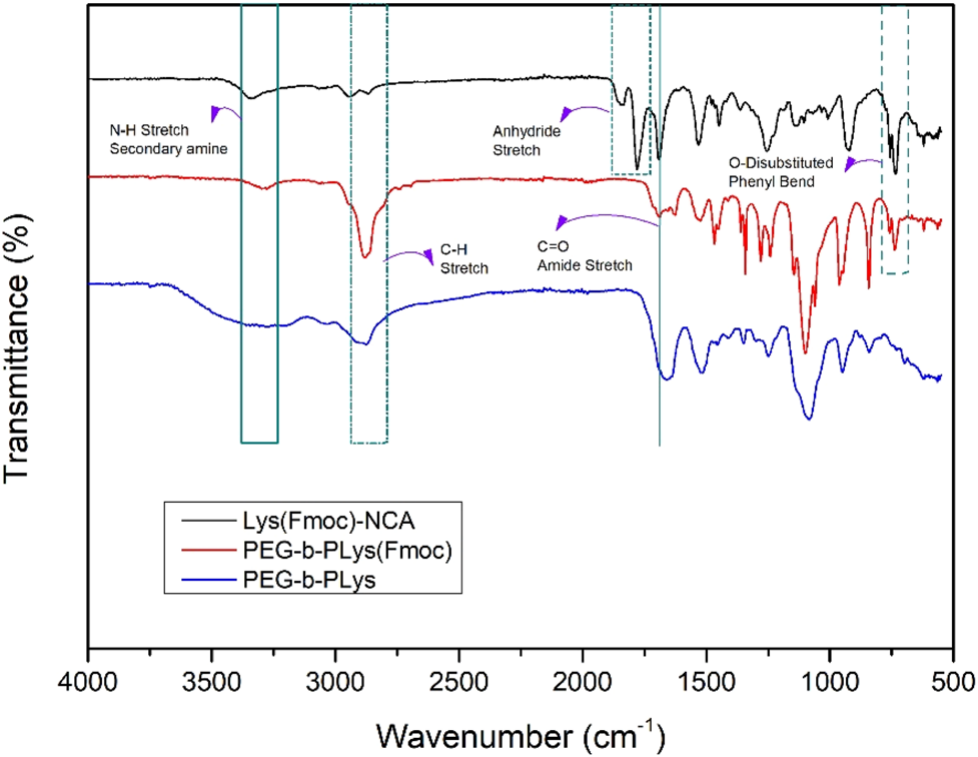
FTIR spectra corresponding to Lys(Fmoc)-NCA, PEG-*b*-PLys(Fmoc), and PEG-*b*-PLys.

FTIR spectroscopy suggested successful block copolymer synthesis owing to the disappearance of the peaks at 1842 cm^−1^ and 1780 cm^−1^ of the NCA that disappear upon complete ring-opening (Fig. 2). The benzyl section of the Fmoc group is represented by peaks at 757 cm^−1^ and 735 cm^−1^.

^1^H NMR spectroscopy further confirmed polymer synthesis (Fig. S4†). The peaks representing the four protons of the ethylene glycol repeat units are represented at 3.55–3.45 ppm, and the terminal methyl protons of the mPEG section are present at 3.24 ppm.^41^ Additionally, peaks representative of the PLys(Fmoc) block can be seen; the peaks between 7.00 and 8.00 ppm are attributed to the aromatic protons of the Fmoc group. The protons of the alkyl chain of the lysine R group are shown between 1.00 and 2.00 ppm. NMR analysis was conducted after several washing steps with different organic solvents to ensure that the peaks present are representative of the desired block copolymer. Integration of the peak corresponding to the terminal methyl protons of PEG *versus* the peak corresponding to the Fmoc aromatic protons revealed that ∼67 Lys(Fmoc) were conjugated to mPEG, on average. APC analysis revealed that a block copolymer with a number average molecular weight (*M*_n_) of 26 500 Da and a dispersity of 1.06 had been produced.

### 2.2 Thermal cleavage of Fmoc to yield PLys

**Fig. 3:**
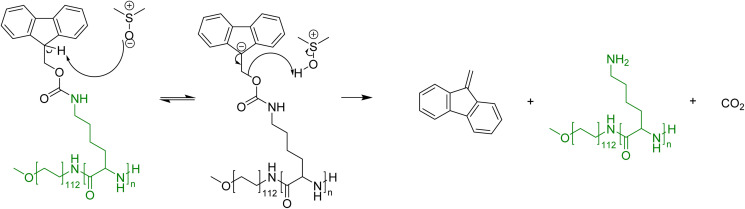
Fmoc cleavage from mPEG-*b*-PLys(Fmoc) in DMSO at elevated temperatures.

The focus of this research is to determine whether the thermally induced cleavage of Fmoc protecting groups can be applied to polymers that possess pendant Fmoc protecting groups. The thermal deprotection of small amino acids is reported to proceed *via* an E1_CB_ mechanism,^35,42^ with DMSO used as the solvent (Fig. 3). It is envisaged that upon heating, the Fmoc groups will decompose to dibenzofulvene and carbon dioxide, leaving amine groups that may be utilised for metal scavenging. This concept has been proven for Fmoc cleavage from small molecules but not Fmoc cleavage from homopolymers, such as PLys(Fmoc)_47_, and block copolymers, such as mPEG_112_-*b*-PLys(Fmoc)_67_.

**Fig. 4:**
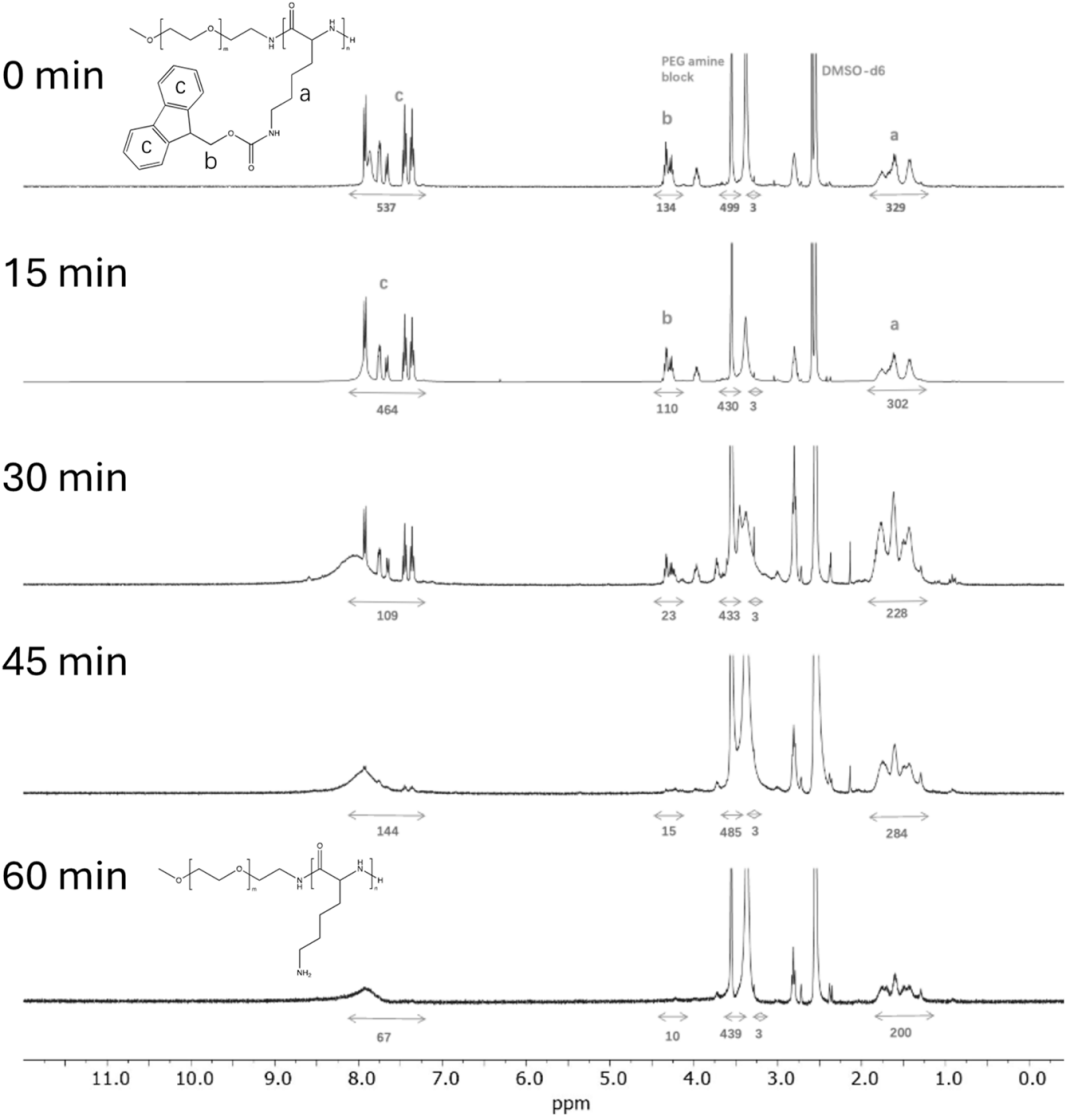
Thermal deprotection process of PEG-*b*-PLys(Fmoc). All spectra were recorded at 400 MHz using DMSO-*d*_6_ as the solvent.

**Fig. 5:**
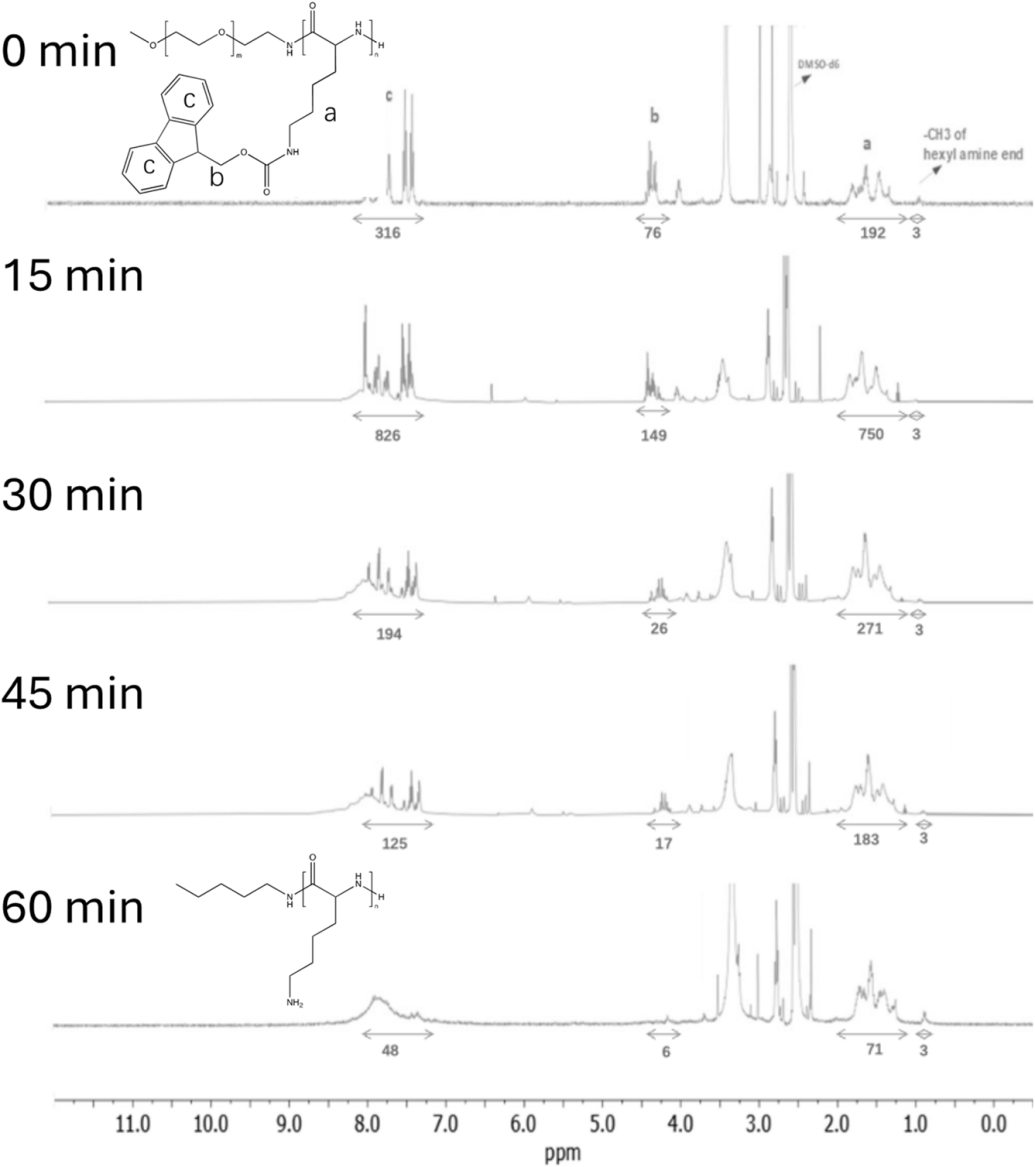
Thermal deprotection of PLys(Fmoc). All spectra were recorded at 400 MHz using DMSO-*d*_6_ as the solvent.

Studies were conducted by independently heating DMSO solutions containing PEG-*b*-PLys(Fmoc) or PLys(Fmoc), and monitoring Fmoc cleavage as a function of time by ^1^H NMR spectroscopy. Fmoc cleavage results in the decrease in the integrals of the protons corresponding to the Fmoc units (peaks b and c in Fig. 4), relative to those of the PLys main chain (peak a in Fig. 4). For PEG-b-PLys(Fmoc), differences in the ^1^H NMR spectra at 0 minutes and 15 minutes were subtle, but the ratio of peak b: peak a slightly decreased from 0.41 to 0.37 and the ratio of peak c: peak a decreased from 1.63 to 1.54 (Fig. 5). Values of 0 for both ratios signify complete Fmoc deprotection. After 30 minutes, the peaks corresponding to Fmoc protons became significantly less sharp and a broad peak above 8 ppm gradually appeared, attributed to the protons of a newly formed primary amine group. There was a sharp decrease in the values for the peak b: peak a ratio (0.10) and the peak c: peak a ratio (0.48), signifying extensive Fmoc removal in both cases. At 60 minutes, the Fmoc groups could not be detected, signifying their complete removal. The broad peak above 8 ppm is retained signifying primary amine liberation.

Fmoc cleavage from PLys(Fmoc) homopolymer was somewhat retarded compared to Fmoc cleavage from mPEG-*b*-PLys(Fmoc), with significant Fmoc removal not observed until 30 minutes (Fig. 4 and 5). The hydrophilic PEG section may aid block copolymer dispersion, enabling increased access to Fmoc groups in comparison to the homopolymer where intermolecular interactions likely cause tight Fmoc group packing. After 30 minutes the peaks corresponding to the Fmoc protons became less sharp and a broad peak signifying the presence of a primary amine formed. The peak b: peak a ratio significantly decreased from 0.40 to 0.10 and the peak c: peak a ratio reduced by 50%, signifying the loss of Fmoc groups. After 60 minutes almost complete Fmoc deprotection was achieved, based on the near complete loss of peaks corresponding to Fmoc protons in the ^1^H NMR spectrum.

APC revealed that the number average molecular weight value (*M*_n_) for PEG-*b*-PLys(Fmoc) was 24 800 Da, signifying that ∼57 Lys(Fmoc) units were attached to the PEG block (Table 1). This value was slightly less than the results provided by ^1^H NMR spectroscopy (∼67 units), although this is not unexpected as the APC value is obtained against PEG homopolymer standards. PLys(Fmoc) was created to ∼43 repeat units which aligned closely to the value provided by ^1^H NMR spectroscopy (∼40 repeat units). Following thermal deprotection, ∼52 (PEG-*b*-PLys) and ∼43 repeat units (PLys) were found on average for each polymer by APC, confirming that polymer backbone hydrolysis had not occurred. The dispersity values of the polymers produced are close to 1 in most cases, signifying narrow molecular weight distributions.

**Table 1:** Molecular weight of protected and deprotected versions of PEG-b-PLys(Fmoc) and PLys. *M*_w_ signifies the weight average molecular weight, *M*_n_ signifies the number average molecular weight

Sample	*M*_W_ (Da)	*M*_n_ (Da)	Dispersity
PLys(Fmoc)	16 000	15 000	1.06
PLys	6700	6000	1.12
PEG-b-PLys(Fmoc)	26 500	24 800	1.06
PEG-*b*-PLys	12 000	11 400	1.05

### 2.3 Pb^2+^ removal from aqueous solution

Biodegradable and bioderived polymers that are water-soluble have numerous potential applications, including drug delivery systems,^43,44^ wound dressing,^45^ sustainable packaging,^46^ wastewater treatment.^47^ A further key application of such polymers is their use in wastewater purification, particularly for the removal of cytotoxic metals from aqueous environments. Acidic Pb^2+^-containing wastewater is of great concern owing to its toxicity and the difficulty in its treatment. The removal of Pb^2+^ from the water system is of paramount importance owing to the damage that Pb^2+^ can impart to the heart, kidneys, and reproductive and nervous systems of humans, and in particular children.^48^ Polymers that boast pendant primary amine functionality are reported to be effective for Pb^2+^ adsorption owing to chelation between the electron lone pairs of the primary amine nitrogen atom and the metal cation.^49^ Although at neutral pH, many primary amine groups become protonated and unable to donate electron lone pairs, it may be hypothesised that the high density of amine groups in PLys ensures that some amine groups remain uncharged and/or favour metal binding over protonation. Consequently, the Pb^2+^ adsorption capabilities of PEG-*b*-PLys and PLys from aqueous solution was assessed.

**Fig. 6:**
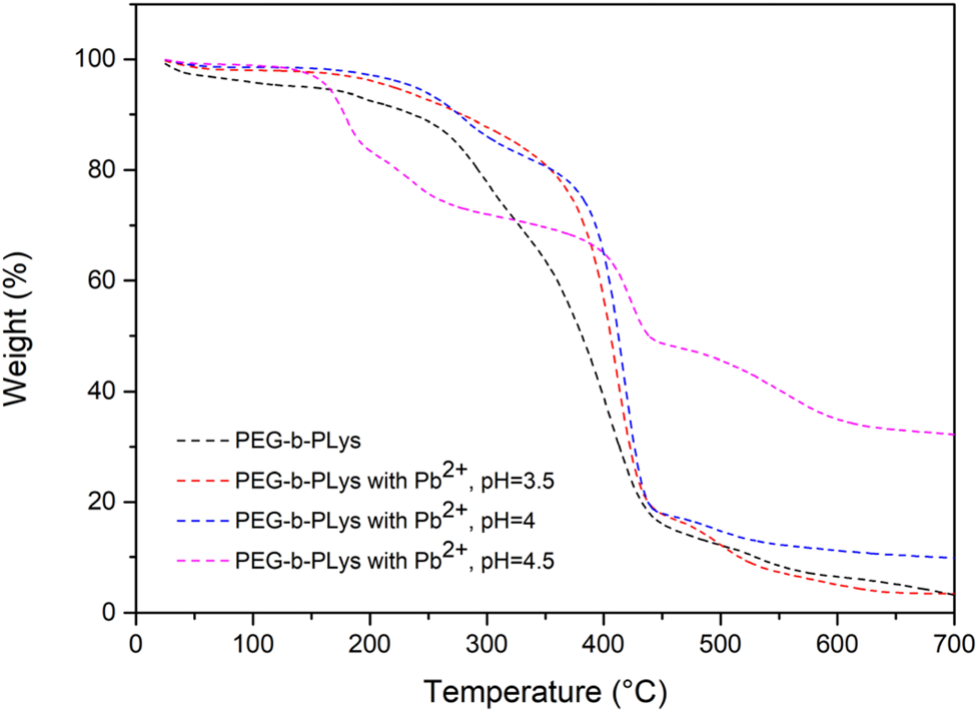
Adsorption of Pb^2+^ by PEG-*b*-PLys from solutions of various pH.

The effectiveness of the polymers produced to interact with and bind Pb^2+^ was assessed using TGA to quantify Pb^2+^ uptake by the polymers. Following polymer-metal incubation and Pb^2+^ recovery, any remaining matter after heating to 700 °C was assumed to be non-organic and thus correspond to Pb^2+^. The amount of Pb^2+^ recovered per 1 g of PEG-*b*-PLys was found to be 2 mg (pH = 3.5), 71 mg (pH = 4) and 408 mg (pH = 4.5) (Fig. 6). Although the extent of Pb^2+^ uptake from pH 4.5 solution highlighted the potential of the materials for the intended application, the difference in Pb^2+^ uptake as the solution pH changes from pH 3.5 to pH 4.5 was somewhat surprising.

**Fig. 7:**
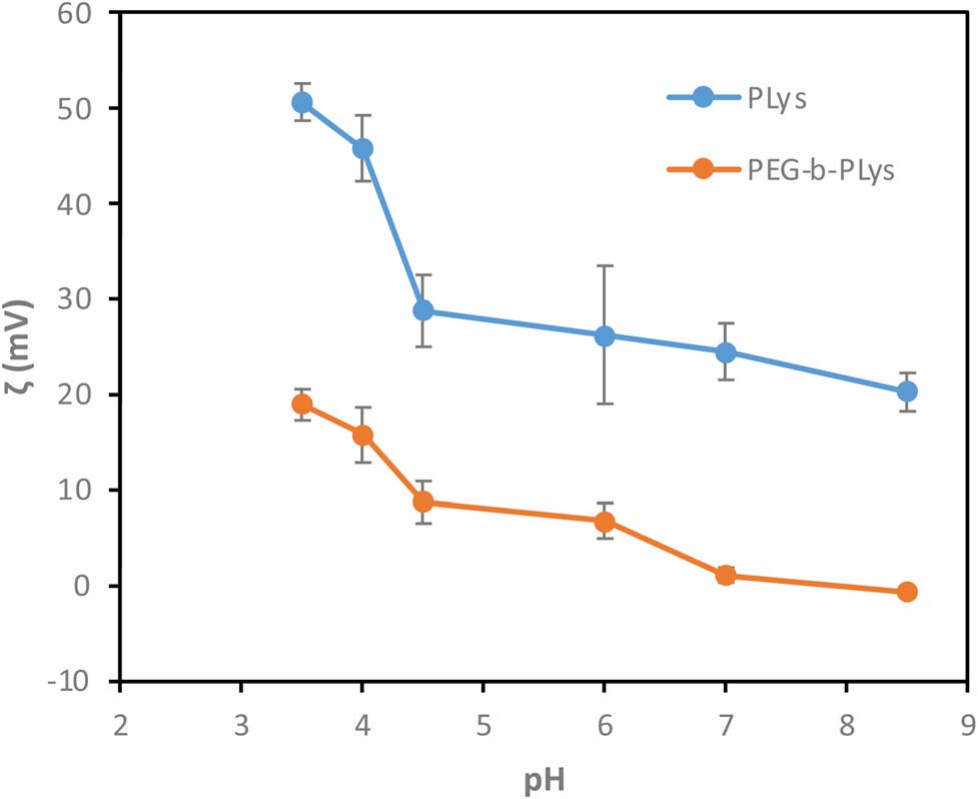
*ζ* potential values for PEG-*b*-PLys and PLys in various pH solutions. The experiments were carried out in triplicate.

Zeta (*ζ*) potential measurements were therefore conducted to determine changes in polymer particle stability caused by the amplification or suppression of charge caused by primary amine (de)protonation (Fig. 7). All *ζ* potential values of PEG-*b*-PLys are positive in acidic and neutral solutions, but decrease as the solution pH increases. Importantly, the *ζ* potential value changes from 19.0 mV to 15.9 mV as solution pH changes from pH 3.5 to pH 4, and a relatively modest value of 8.8 mV is recorded in solution of pH 4.5. A large *ζ* potential value, positive or negative, denotes particle stability which would arise due to electrostatic repulsion between polymer particles in this instance. As the pH increases, cationic charge is supressed rendering polymer aggregation more likely. This suppression of charge due to pendant primary amine deprotonation may feasibly promote Lys-Pb^2+^ chelation rather than Lys-Pb^2+^ repulsion, hence greater Pb^2+^ uptake being recorded in pH 4.5 solution compared to both pH 3.5 and pH 4 solutions.^50^

**Fig. 8:**
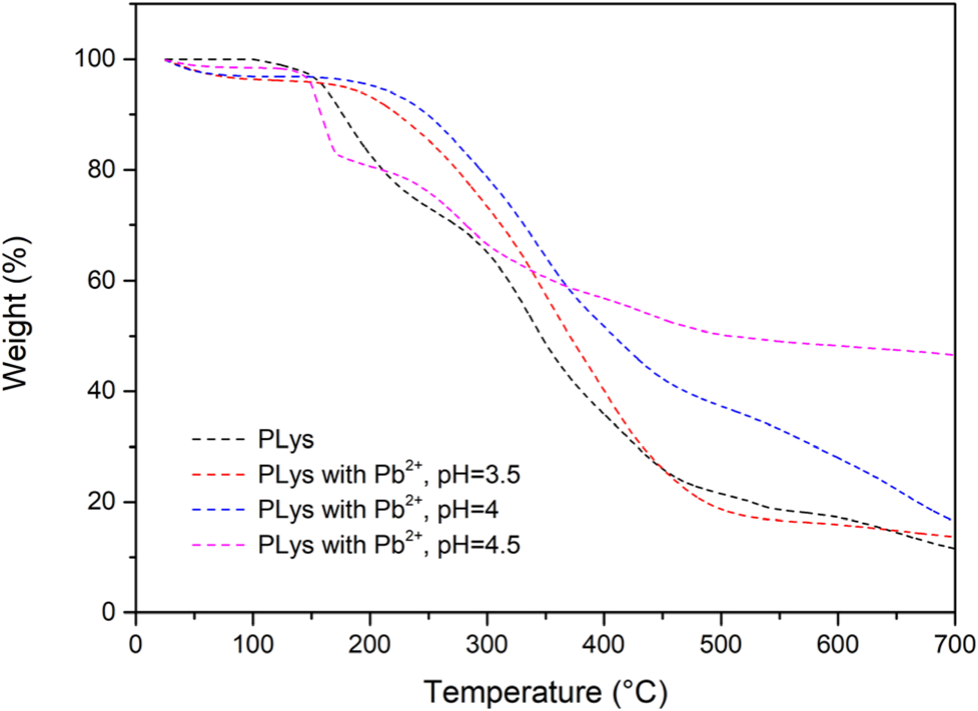
TGA data to represent the adsorption of Pb^2+^ by PLys from solutions of various pH.

PEG was included in PEG-*b*-PLys formulations as hydrophilic PEG should facilitate metal-polymer dispersion in aqueous solution after the binding event. A water-soluble polymer is converted to a dispersed polymer that can easily be recovered by filtration following metal scavenging. However, the PEG block may be redundant as PLys is water-soluble itself. 1 g of polymer was able to adsorb 538 ± 28 mg of Pb^2+^ according to TGA analysis (Fig. 8, experiments carried out in triplicate). As the extent of adsorption exceeded that of PEG-*b*-PLys (408 ± 17 mg of Pb^2+^ per 1 g of PEG-*b*-PLys), the use of PEG was deemed unnecessary. Enhanced adsorption by PLys compared to PEG-*b*-PLys is not unexpected given the increased primary amine loading of the homopolymer relative to the polymer molecular weight, with this study emphasising the significance of PLys as a very useful material in its own right.

Unlike PEG-*b*-PLys, PLys exhibits a positive *ζ* potential values at pH levels as high as 8.5 (Fig. 8). It may be hypothesised that absence of the PEG segment leads to elevated *ζ* potential values in PLys since the introduction of the PEG block may serve to shield the positive charge originating from amine groups.^51,52^ A significant reduction in *ζ* potential values of PLys is observed when pH ranges from 3.5 to 4.5. The value drops to 20.4 mV at pH 4.5, matching the vastly increased amount of adsorbed Pb^2+^ by PLys in aqueous solution of this pH.

A paired *t*-test comparing PLys and PEG-*b*-PLys across the different pH values showed a t-statistic of 11.01 and a *p*-value of 0.00011. This very low *p*-value indicates a highly statistically significant difference between the two groups.

The maximum Pb^2+^ loading capacities of were compared with those from previous studies by poly(amino acids)-based adsorbents (Table 2). However, the preparation of PEG-*b*-PLys and PLys in our studies was done using thermal deprotection as opposed to acid or base deprotection. Besides, PLys can capture more Pb^2+^ from acetic aqueous environment than previous poly(amino acids)-based adsorbents. Therefore, it has the potential to control the accidental Pb^2+^ pollution.

**Table 2:** A comparison of various adsorbents for the removal of Pb^2+^ from the aqueous solution

No.	Adsorbents	pH	Maximum adsorption capacity (mg g^−1^)	Reference
1	γ-Polyglutamic acid grafted lignin	5.5	276	53
2	ε-Poly-l-lysine grafted lignin	5.5	232	53
3	Poly-l-glutamic acids/polypolyethylene–silica complex membranes	5.5	298	54
4	Poly-l-aspartic acids/polypolyethylene–silica complex membranes	5.5	121	54
5	PEG-*b*-PLys	4.5	408 ± 17	This study
6	PLys	4.5	538 ± 28	This study

## 3. Conclusions

This study presents a groundbreaking approach to the synthesis of linear homopolymer PLys and linear block copolymer PEG-*b*-PLys *via* NCA ROP, eliminating the need for toxic deprotection agents for the first time. These water-soluble polymers combine bio-renewability, biodegradability, extensive chemical functionality, and cationic charge, positioning them as valuable candidates for a wide range of industrial and environmental applications. The development of a safe, cost-effective, and efficient thermal deprotection method, achieved within just 1 hour by heating to 120 °C in recyclable DMSO, marks a significant advancement in sustainable polymer production. The demonstrated ability of PLys and PEG-*b*-PLys to adsorb Pb^2+^ from aqueous solutions at capacities of 538 mg g^−1^ and 408 mg g^−1^, respectively, highlights their potential for addressing urgent environmental challenges, such as heavy metal pollution. Future research should expand the application scope of α-polylysine and PEG-*b*-PLys beyond Pb^2+^ ions to other significant heavy metals such as cadmium, mercury, and chromium, to fully exploit their potential in diverse environmental remediation scenarios. Additionally, studying these polymers under representative environmental conditions represents an imminent research goal. However, this study not only reduces the environmental footprint and cost of PLys production but also opens new avenues for their widespread application in water purification, biotechnology, and beyond. By enhancing the safety, scalability, and sustainability of PLys production, this work paves the way for the next generation of functional polymers to make a tangible impact in addressing global sustainability challenges.

## Data availability

The data supporting this article have been included as part of the ESI.†

## Conflicts of interest

There are no conflicts to declare.

## Supplementary Material

RA-015-D5RA00641D-s001
