# Supplementary material for: Thermal deprotection: a sustainable and efficient strategy for synthesising α-polylysine adsorbents
Source: RSC Adv. 2025 May 23;15(22):17397–404. doi: 10.1039/d5ra00641d (PMC12101115; doi:10.1039/d5ra00641d)
Supplement: RA-015-D5RA00641D-s001 [file RA-015-D5RA00641D-s001.pdf]

Supplementary Information (SI) for RSC Advances.
This journal is © The Royal Society of Chemistry 2025

Supplementary information for:

Thermal Deprotection: A Sustainable and Efficient Strategy for Synthesising α-Polylysine Adsorbents

Xuchen Jin and Paul D. Thornton*

* p.d.thornton@leeds.ac.uk

Materials

Fmoc-Lys-OH was obtained from Flurochem Incorporation. Triphogene (98%), (-)-α-Pinene, hexane (97%), PEG amine (Mn=5000), hexyl amine, dialysis tubes (2000Da) and phosphate buffered saline tablets were purchased from Sigma–Aldrich Corporation. Dimethyl sulfoxide (DMSO)-d_6 (99.8% D) was obtained from EURISO-TOP. Dimethylformadime (DMF) (99.5%), DMSO, diethyl ether (99.5%) and lead (II) chloride were purchased from Fisher Scientific International Incorporation. All reagents were used as purchased.

Synthesis Methods

Preparation of Lys(Fmoc)-NCA

As a general procedure, 16 mmol of Fmoc-Lys-OH was added into a three-necked round bottom (RB) flask connected a condenser, nitrogen supply, and a dropping funnel. The flask with this amino acid was maintained under vacuum for an hour to eliminate water from the system, and then 70 mL of anhydrous tetrahydrofuran (THF) and alpha-pinene (32 mL, 32 mmol) were added to the RB flask under a steady flow of nitrogen. 10 g (33mmol) triphosgene dissolved in 20 mL anhydrous THF was added to the reaction mixture dropwise through the dropping funnel until the heated mixture began to reflux. The reaction was complete when the mixture became clear and transparent solution, which demonstrates the generation of Lys(Fmoc)-NCA that is soluble in THF. The whole reaction last 6 h. If reaction mixture remained cloudy, the clear liquid was collected by filtration. The clear solution created was rotary evaporated to ~20 mL before precipitation into hexane yielded the NCA (hexane: THF ratio = 8:1, v/v). This was stored in a freezer overnight to facilitate further NCA precipitation. The crude product was collected by vacuum filtration and washed with

cold THF to remove the unreacted reagents. The final product was obtained under vacuum at room temperature (RT) after at least twice recrystallisation processes. ^{1}H NMR (400 MHz, DMSO-d6): δ 9.10 (s, 1H), 7.94-7.31 (m, 8H), 7.31-7.26 (m, 1H), 4.47-4.39 (m, 1H), 4.33-4.26 (m, 2H), 4,25-4.18 (m, 1H), 3.03-2.94 (m, 2H), 1.78-1.59 (m, 2H), 1.49-1.10 (m, 4H); FTIR: Vmax/cm^{-1} (solid): 3341 (N-H), 3066 (C-H from phenyl), 1842 (C=O), 1780 (C=O), 1690 (C=O from acylamino), 757 and 735 (benzyl ring). Yield:63.8%

Synthesis of PLys(Fmoc)

Lys(Fmoc)-NCA was added to an oven dried Schlenk tube that was purged with nitrogen for an hour. Anyhdrous DMF was added and the solution underwent mixing prior to a solution of hexylamine dissolved in DMF being added by syringe. Anhydrous conditions were maintained during four days of polymerisation conducted at room temperature. Polymer recovery was achieved by adding the reaction solution dropwise to stirred, ice cold, diethyl ether, prompting polymer precipitation. The solid polymer was then isolated from the liquid by centrifugation. This process was repeated twice before the final polymer product was washed several times with diethyl ether and dried in the vacuum oven at 55 °C overnight. ^{1}H NMR (400 MHz, DMSO-d6): 7.93-7.17 (m, 8H), 4.40-4.15 (m, 2H), 1.80-1.04 (m, 6H), 0.90-0.74 (t, 3H).

Synthesis of PEG-*b*-PLys(Fmoc)

Synthesis of the amphiphilic block copolymer PEG-*b*-PLys(Fmoc) was conducted in a comparable manner to the synthesis of PLys(Fmoc), but with the hexylamine initiator being replaced with a PEG_{5000} macroinitiator, where 5,000 denotes the polymer number average molecular weight. Lys(Fmoc)-NCA (300 mg, 0.79 mmol) was dissolved in DMF (3 mL) and added to an oven dried Schlenk tube. The macroinitiator, methoxyPEG-amine (65 mg, 0.013 mmol) was dissolved in 3 mL DMF with the aid of sonication (5 minutes) and added to Schlenk tube. Polymerisation was undertaken under nitrogen at room temperature for four days. Polymer recovery was achieved by adding the reaction solution dropwise to stirred, ice cold, diethyl ether, prompting polymer precipitation. The solid polymer was then isolated from the liquid by centrifugation. This process was repeated twice before the final polymer product was washed several times with diethyl ether and dried in the vacuum oven at 55 °C

overnight. ^{1}H NMR (400 MHz, DMSO-d6): 7.98-7.15(m, 8H), 4.38-4.03 (m, 2H), 3.56-3.42 (m, PEG), 3.24 (s, 3H, PEG),1.80-1.12 (m, 6H).

Thermal deprotection of PEG-b-PLys(Fmoc) and PLys(Fmoc)

The Fmoc protected (block co-)polymer (200 mg) was dissolved in DMSO (6 mL), and the solution stirred at 120 °C. To monitor the deprotection kinetics, 1 mL of solution was retrieved from the solution every 15 minutes by syringe, and the polymer recovered by precipitation into ice cold diethyl ether. Polymer isolation from solvent was done by centrifugation, with the retrieved solid washed several times with ice cold diethyl ether before being dried overnight in a vacuum oven at 55 °C. Larger samples used for Pb^{2+} adsorption were created over 1 h but without the intermittent sampling.

Pb^{2+} Adsorption by PEG-b-PLys and PLys

50 mg $PbCl_2$ was dissolved in DI water (10 mL). 20 mg of PEG-*b*-PLys or PLys was mixed with Pb^{2+} solutions at pH 3.5, 4 and 4.5. The polymer under investigation was added (20 mg) to the solution and the mixture was maintained for 1 day at room temperature. Following this, each sample underwernt dialysis against deionised water for two days with frequent water changes to remove non-adsorbed Pb^{2+}. The remaining material was recovered by lyophilisation.

<u>Characterisation Methods</u>

Fourier-transform infrared (FTIR) spectroscopy

All samples were stored under vacuum oven at 55 °C for at least 1 day prior to FTIR analysis. All spectra were recorded via Perkin Elmer Spectrum One equipped with Bruker OPUS 7.0 software and a Specac Golden Gate Attenuated Total Reflection (ATR) diamond top plate. 100 scans were taken and the vibrational frequencies displayed in cm^{-1}.

^{1}H Nuclear magnetic resonance (NMR) spectroscopy

^{1}H NMR spectra was tested by a Bruker AV3HD 400MHz NMR spectrometer equipped with a 5 mm BBO Probe. Chemical shifts (ppm) were calibrated using trimethylsilane (TMS), the chemical shift of which is 0 ppm. XR-55 NMR tubes (Norell) were used. NMR spectra were analysed using MestreNova x64. Abbreviations used in ^{1}H NMR analysis are: singlet (s), doublet (d), triplet (t), multiplet (m), doublet of doublets (dd). $CDCl_3$ and DMSO-d6 were used as the deuterated solvents.

Advanced polymer chromatography (APC)

Average polymer molecular weight was deteremined by APC, a form of size exclusion chromatography. Specifically, an ACQUITY APC system, equipped with an ACQUITY refractive index (ACQ-RI) detector was used. The column temperature was maintained at 40 °C and the flow rate at 0.5 mL/minute. System calibration was carried out using PEG standards and data processed using Empower 3 software. DMF containing 1 g/L LiBr was used as the mobile phase on an ACQUITY APC AQ column packed with bridged poly(ethylene) hybrid particles (200Å, 2.5 µm).

Thermalgravimetric analysis (TGA)

The polymers and polymer-Pb^{2+} complexes were analysed by TGA (TA Instruments TGA Q50 model) to determine the adsorbed Pb^{2+} content. The apparatus was continually flushed with oxygen and temperature increased from RT to 700 °C at a rate of 10 °C/min. All experiments were performed in triplicate.

Zeta (ζ) potential measurements

20 mg samples were totally dissolved in the 10 mL NaCl solution (concentration:10 mM) firstly. Then the pHs of the solutions were adjusted to the desired values with 0.05 M HCl or 0.05 M NaOH. ζ potential measurement was conducted using a Malvern Zetasizer Nano ZSP instrument (Malvern Panalytical Ltd, Malvern, UK). All experiments were performed in

triplicate, with each data point collected the average of five measurements by the software.

Supplementary figures

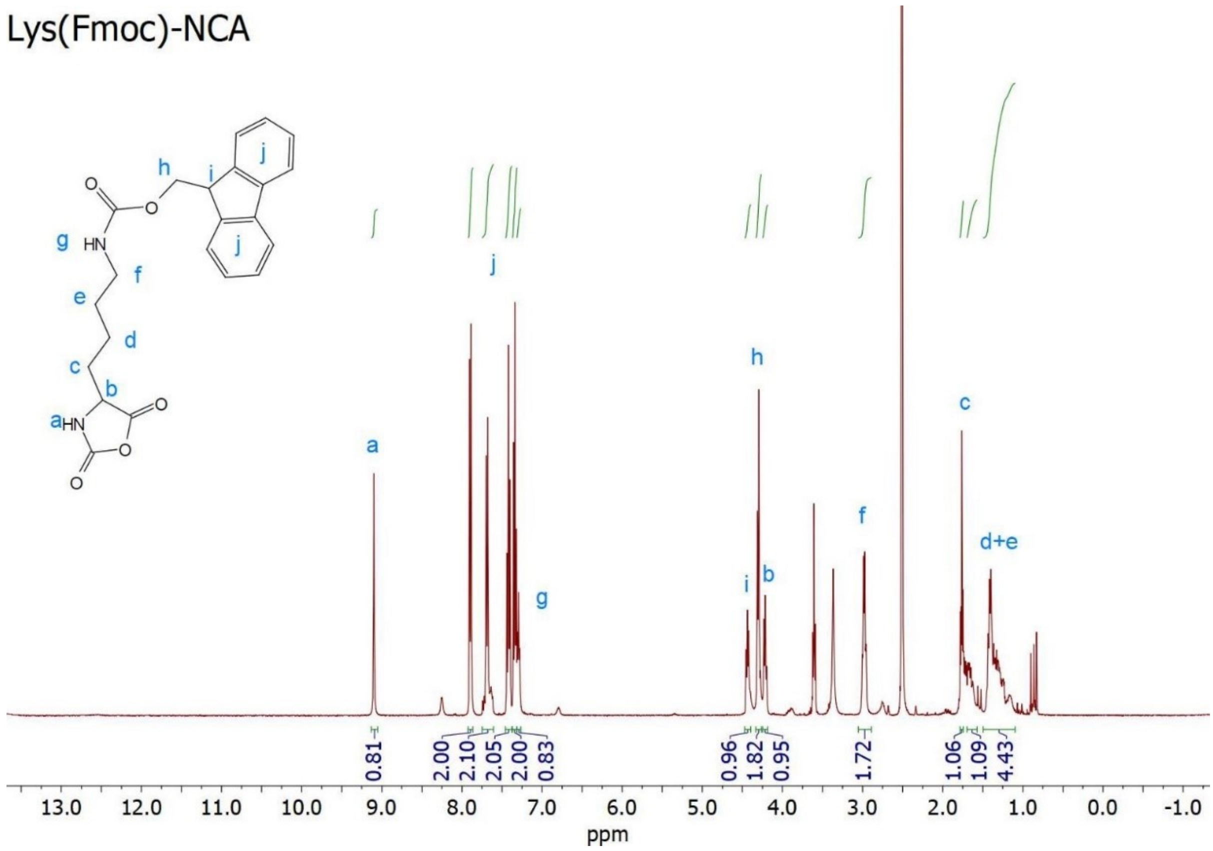

Fig.S1 ^{1}HNMR spectrum of Lys(Fmoc)-NCA recorded at 400 MHz. DMSO-d6 was used as the solvent.

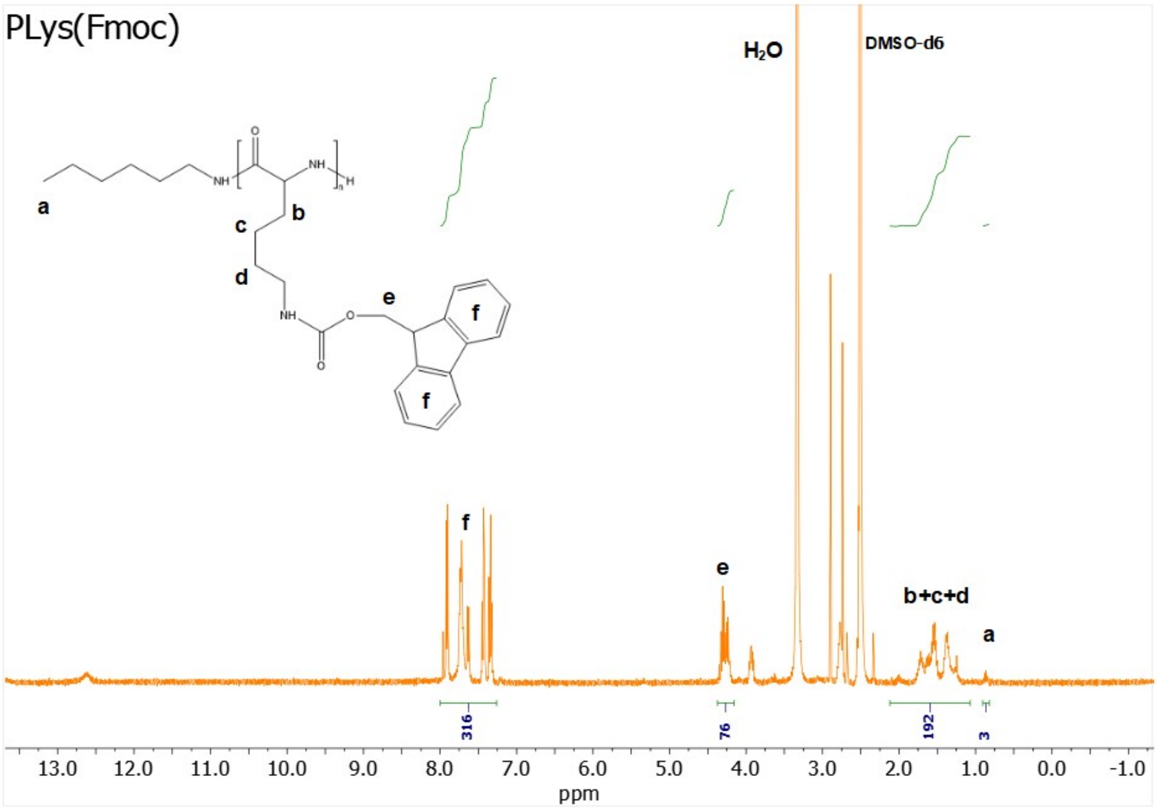

Fig.S2 ^{1}HNMR spectrum of PLys(Fmoc) recorded at 400 MHz. DMSO-d6 was used as the solvent.

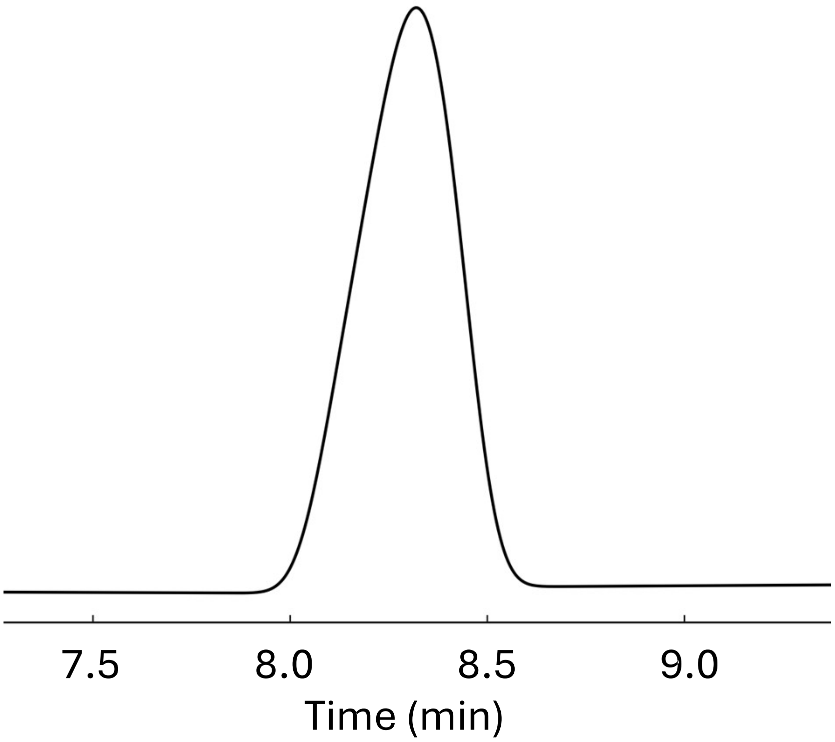

Fig S3. APC chromatogram curve corresponding to the elution of PLys(Fmoc).

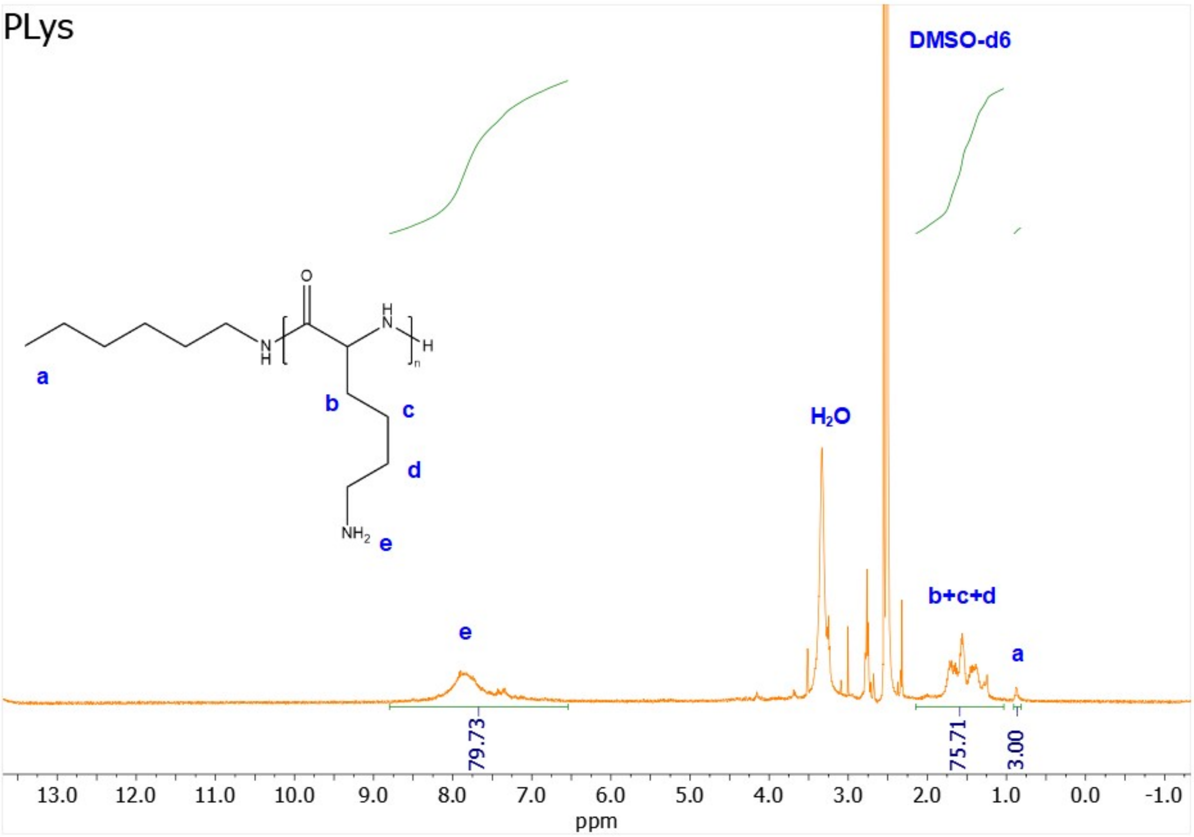

Fig.S4. ^{1}HNMR spectrum of PLys recorded at 400 MHz. DMSO-d6 was used as the solvent.

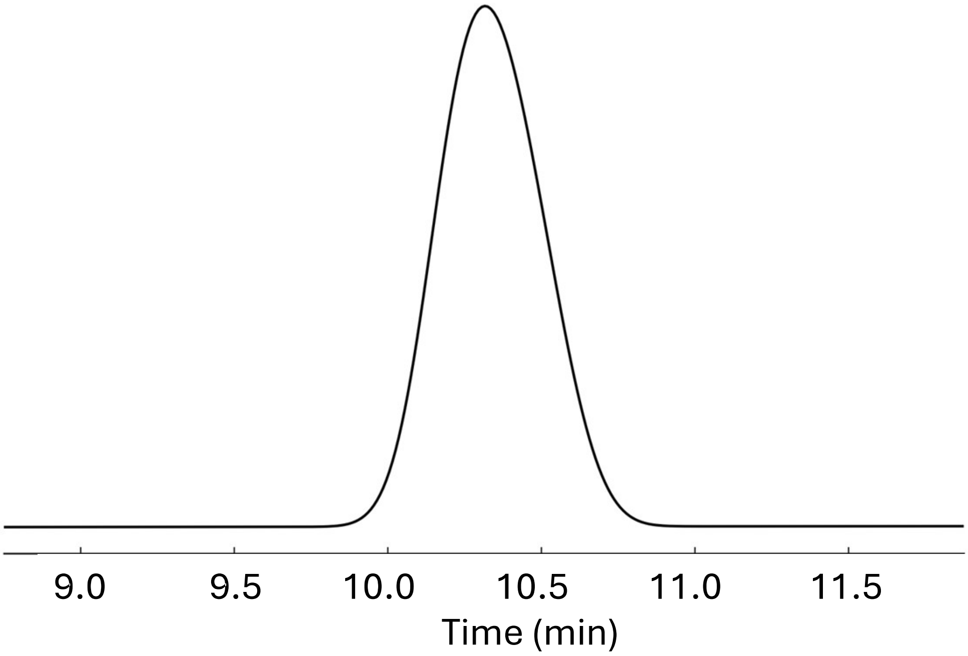

Fig.S5. APC chromatogram curve corresponding to the elution of PLys.

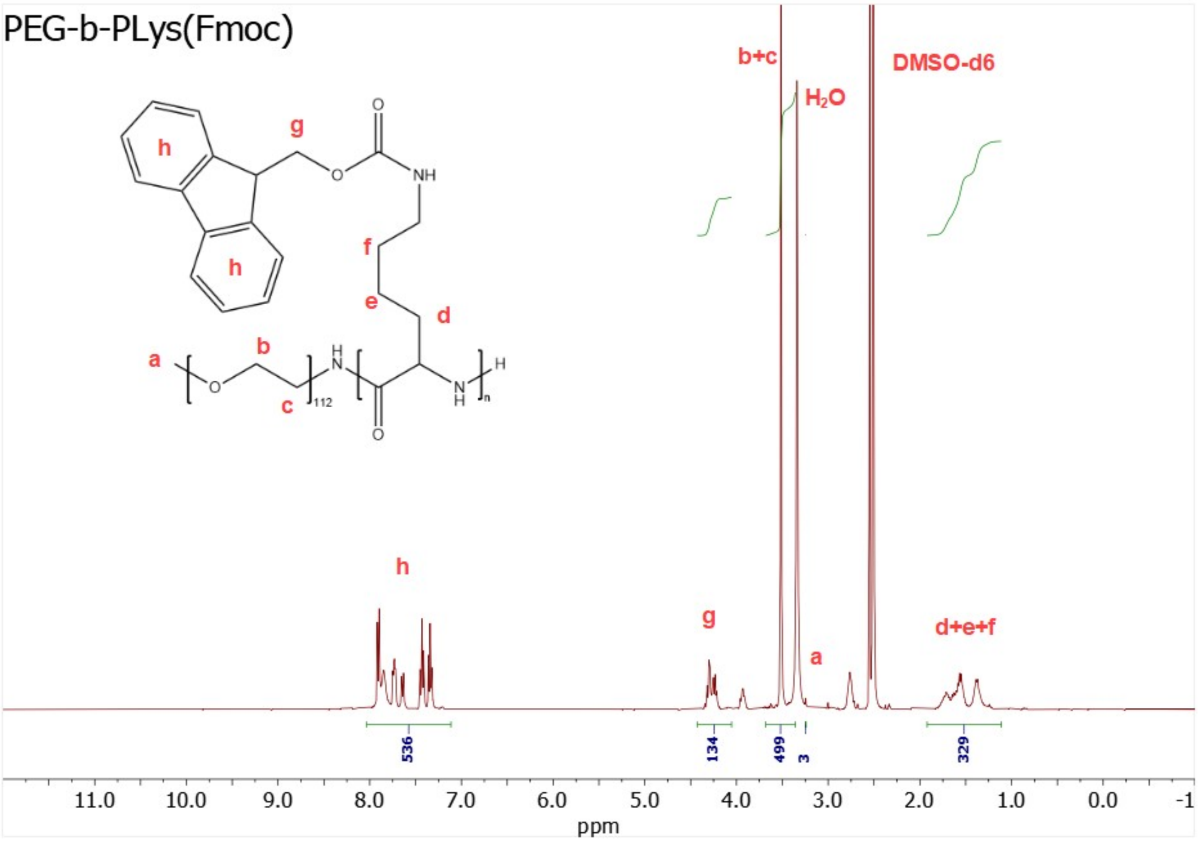

Fig.S6. ^{1}HNMR spectrum of PEG-*b*-PLys(Fmoc) recorded at 400 MHz. DMSO-d6 was used as the solvent.

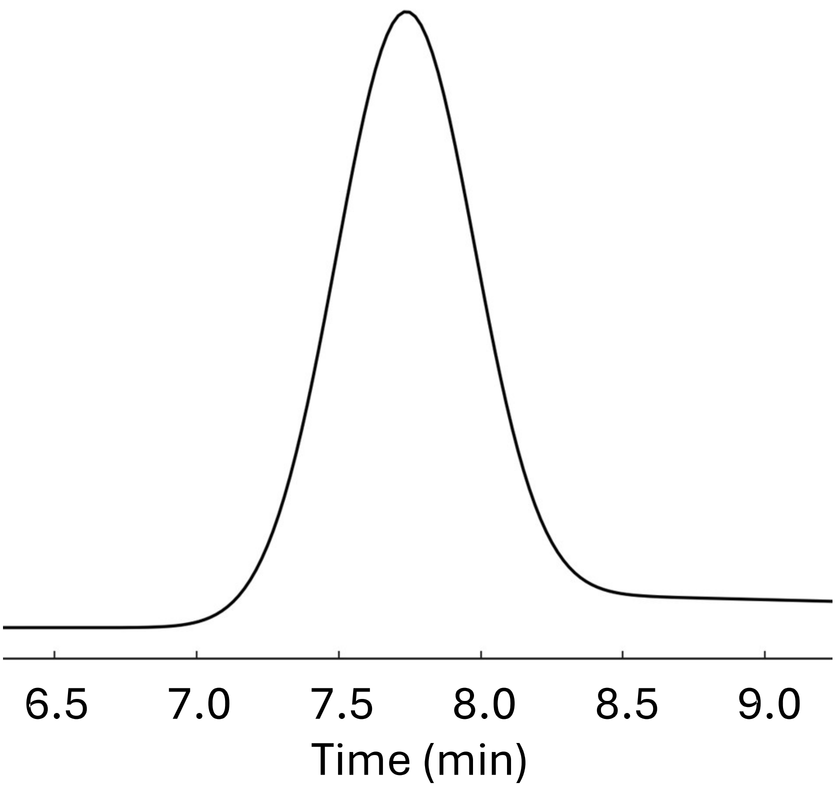

Fig.S7. APC chromatogram curve corresponding to the elution of PEG-*b*-PLys(Fmoc).

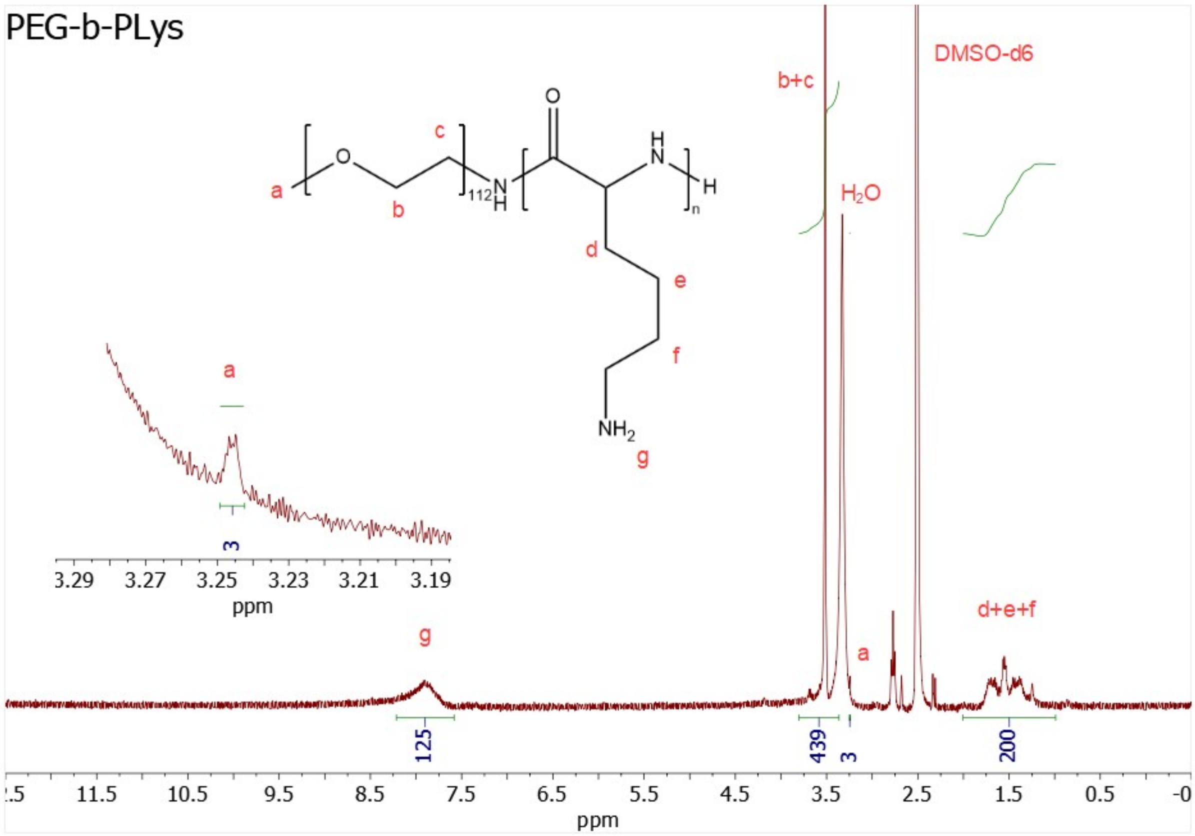

Fig.S8. ^{1}H NMR spectrum of PEG-b-PLys recorded at 400 MHz. DMSO-d6 was used as the solvent.

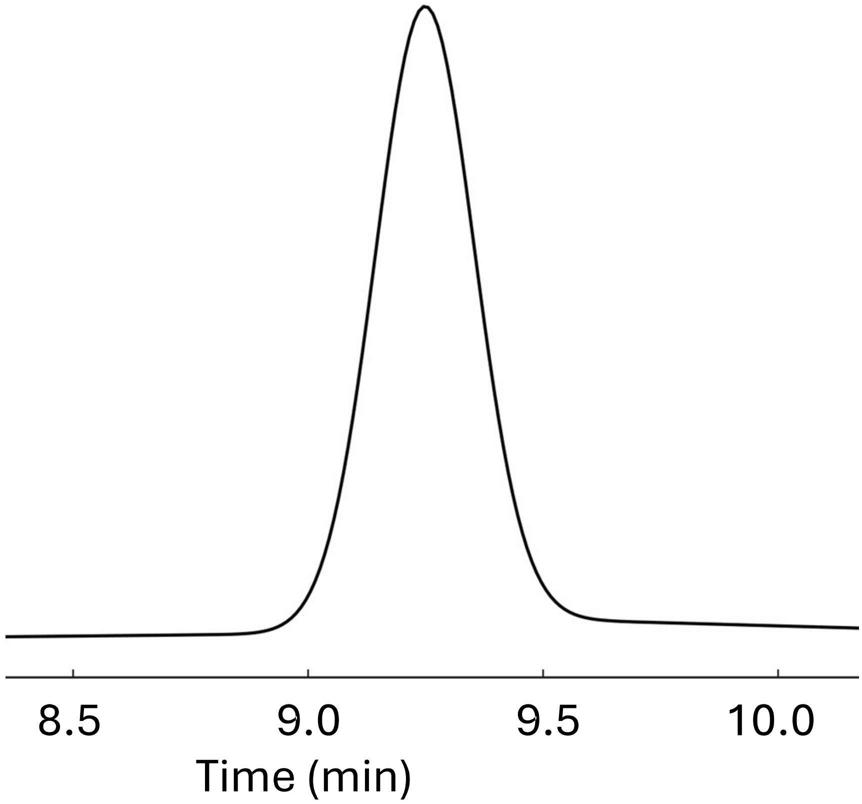

Fig.S9. APC chromatogram curve corresponding to the elution of PEG-*b*-PLys.
